# Development Of Diet Score For Reflecting Diet Patterns and Dietary Guidelines: A Case Of Singapore

**DOI:** 10.1093/geroni/igab046.3055

**Published:** 2021-12-17

**Authors:** Yuezhong Liu, Rakhi Verma, Ringo Ho, Yin-Leng Theng

**Affiliations:** 1 Nanyang Technological University, Singapore, Singapore; 2 Nanyang Technological University, Singapore; 3 Nanyang Technological University, Singapore, Singapore, Singapore

## Abstract

Foods and dietary patterns substantially affect health outcomes. The overall dietary assessment score associated with dietary guidelines in Singapore has not been assessed previously. This study aimed to develop and evaluate diet score for identifying the relationship between dietary patterns and dietary guidelines in Singapore. Using a localised diet score survey collaborated with the Commonwealth Scientific and Industrial Research Organisation (CSIRO), we conducted a cross-sectional study of 600 Singapore persons in two-generational cohorts (40-64: 300 and > 65 years: 300). The proposed local diet score was calculated to reflect their overall compliance with the Dietary Guidelines in Singapore. ANOVA analysis was used to identify the significant difference among socio-demographic variables associated with diet score and comparison analysis was performed to compare the diet patterns and diet score. There are significant differences among age, education, housing, residency associated with diet score. Diet score of older cohort (M= 67.71, SD= 13.38) is significantly higher than young cohort (M= 60.73, SD= 14.71). The highest education level (University or tertiary) obtain the lowest diet score (M= 58.58, SD= 14.41). The participants who live in the landed property (M= 69.45, SD= 14.43) are higher than those who live in Condominium and Public House. And the participants who live alone (M= 67.26, SD= 14.66) have a higher average diet score. Two-generational cohorts are not compliant with recommendations about dietary guideline well in Singapore. The present findings suggest that dietary patterns need improvement in aspects such as vegetables and extra food components.

